# Enzyme-Assisted Ultrasonic Extraction of a Polysaccharide-Rich Extract from *Abelmoschus manihot* (L.) Root and Preliminary Evaluation of Hair Care-Related Properties

**DOI:** 10.3390/molecules31162817

**Published:** 2026-08-13

**Authors:** Junjie Wang, Xueyan Liu, Dian Zhuang, Zixuan Ren, Weihong Chen, Mingqiong Guo, Zenglai Xu, Qiong Wang

**Affiliations:** 1 Department of Chemistry, School of Science, Xihua University, Chengdu 610039, China; 2 Jiangsu Key Laboratory for Conservation and Utilization of Plant Resources, Institute of Botany, Jiangsu Province and Chinese Academy of Sciences (Nanjing Botanical Garden Memorial Sun Yat-Sen), Nanjing 210014, China; 3 Nanjing University of Chinese Medicine, Nanjing 210023, China

**Keywords:** *Abelmoschus manihot* root, enzyme-assisted ultrasonic extraction, polysaccharide-rich extract, hair care efficacy, hair combability

## Abstract

*Abelmoschus manihot* (L.) flowers are widely used in traditional Chinese medicine, whereas the roots are often discarded as agricultural waste. The compact cellular and tissue architecture of the roots hinders the release of intracellular metabolites, thus impeding their reutilization. Here, enzyme-assisted ultrasonic extraction was developed to obtain a polysaccharide-rich crude extract from *A. manihot* roots. Orthogonal optimization identified pH 4.5, a temperature of 60 °C and a cellulase dosage of 6% (w/w) as the optimal extraction conditions, giving an extraction yield of 21.59%. The phenol–sulfuric acid assay indicated a total carbohydrate content of 75.09% (glucose equivalents). Structural analysis by Fourier transform infrared (FT−IR) confirmed the presence of characteristic polysaccharide functional groups, while monosaccharide composition analysis via high-performance liquid chromatography (HPLC) revealed the major components of glucose, glucuronic acid, rhamnose, galacturonic acid, etc. Inspired by the traditional use of *A. manihot* extract in papermaking, its preliminary hair care-related properties were further evaluated. At a concentration of 30 mg/mL, the extract solution inhibited *Malassezia furfur* growth by 84%, reduced wet and dry combing work by 16.93% and 30.86%, and increased tensile strength and tensile fracture energy by 13.04% and 14.84%, respectively. Scanning electron microscope (SEM) images suggested smoother hair fiber surfaces after treatment. These results highlight the extract’s great potential for hair care cosmetic products.

## 1. Introduction

Obtaining natural products from plants not only provides important resources for industrial applications, but also advances the utilization of plant biomass [1,2,3]. *Abelmoschus manihot* (L.) is a flowering plant in the *Malvaceae* family, that is distributed across the tropical to subtropical regions of Asia [4]. Its flowers contain abundant bioactive compounds such as quercetin, isoquercetin and hyperoside [4,5,6], and have been widely used in kidney-related and anti-inflammatory applications [7,8,9].

By contrast, *A. manihot* roots are generated in large quantities after flower harvesting but remain underutilized [10]. To achieve the reutilization of *A. manihot* roots, our previous work obtained a polysaccharide-rich extract from the roots, and explored its skin care efficacy [11]. However, compared to floral cells, the thickened secondary cell walls and multilayered tissue architecture in root cells significantly impede the release of intracellular secondary metabolites [12,13], resulting in reduced extraction yield under normal conditions [14]. Although introducing acidic or alkaline hydrolysis can promote the cell wall degradation and enhance the dissolution of target extracts, these methods may also lead to the degradation of the extracted products [15]. Therefore, developing an efficient and mild extraction method to improve the extraction efficiency from the roots is highly desirable for the valorization of *A. manihot* roots.

Enzyme-assisted ultrasonic extraction (EAUE) has attracted considerable attention due to multiple advantages, including mild operating conditions, enhanced extraction efficiency, reduced energy consumption and improved environmental sustainability [16,17,18]. In general, enzymes can efficiently degrade the cellulose and pectin that constitute the cell wall structure, thereby facilitating the release of intracellular compounds [19,20]. Meanwhile, the cavitation effect induced by ultrasound can produce localized high pressure in solution, which further disrupts the cell wall and enhances the dissolution of intracellular components into the extraction solvent [21,22]. Therefore, EAUE represents a promising approach for improving the extraction yields of polysaccharide-rich extracts from *A. manihot* root residues.

In addition, *A. manihot* root extract was traditionally used as an anti-adhesion and dispersion aid (*Zhiyao*) in Chinese papermaking [23]. This extract is rich in polysaccharides that form protective layers on paper fiber surfaces and maintain a homogeneous fiber suspension, thereby promoting pulp lubrication and paper formation [24]. This historical use motivated the hypothesis that *A. manihot* root extract could also deposit on hair fibers, reduce surface friction, and improve combability, thereby highlighting its potential as a natural additive in hair care cosmetic products.

Herein, an EAUE method was developed to achieve high-efficiency extraction of a polysaccharide-rich extract from *A. manihot* roots. The key extraction conditions including enzyme type, pH, extraction temperature and time were systematically examined, and the main extraction factors were optimized using an orthogonal design. The obtained crude extract was characterized by Fourier transform infrared (FT-IR) spectroscopy, thermogravimetric analysis (TGA) and monosaccharide composition analysis of its acid hydrolysate. The preliminary hair care efficacies including anti-dandruff efficacy, tensile performance, and combability were subsequently assessed. This work not only demonstrates the value of EAUE for valorizing agricultural residues but also highlights the potential of natural polysaccharide-rich extracts as ingredients in hair care products.

## 2. Results and Discussion

### 2.1. Analysis of Single-Factor Experiments

#### 2.1.1. Effect of Enzyme Type on Extraction Yield

Cellulase, pectinase, and papain were screened because cellulose, pectin, and proteins are essential structural components of root cell walls [25]. At an equal enzyme dosage of 1% (w/w; relative to dry root powder), corresponding to activities of 2.0 × 10^4^ U/g for cellulase, 2.5 × 10^4^ U/g for pectinase, and 1.0 × 10^4^ U/g for papain, cellulase exhibited the highest extraction efficiency (15.25 ± 0.15%), followed by pectinase (13.32 ± 0.18%) and papain (12.45 ± 0.31%), and was therefore selected for subsequent optimization. The higher extraction efficiency of cellulase may be attributed to cellulase’s stronger ability to degrade the cell wall structure and facilitate the release of intracellular substances.

#### 2.1.2. Effect of Extraction Time on Extraction Yield

**Figure 1:**
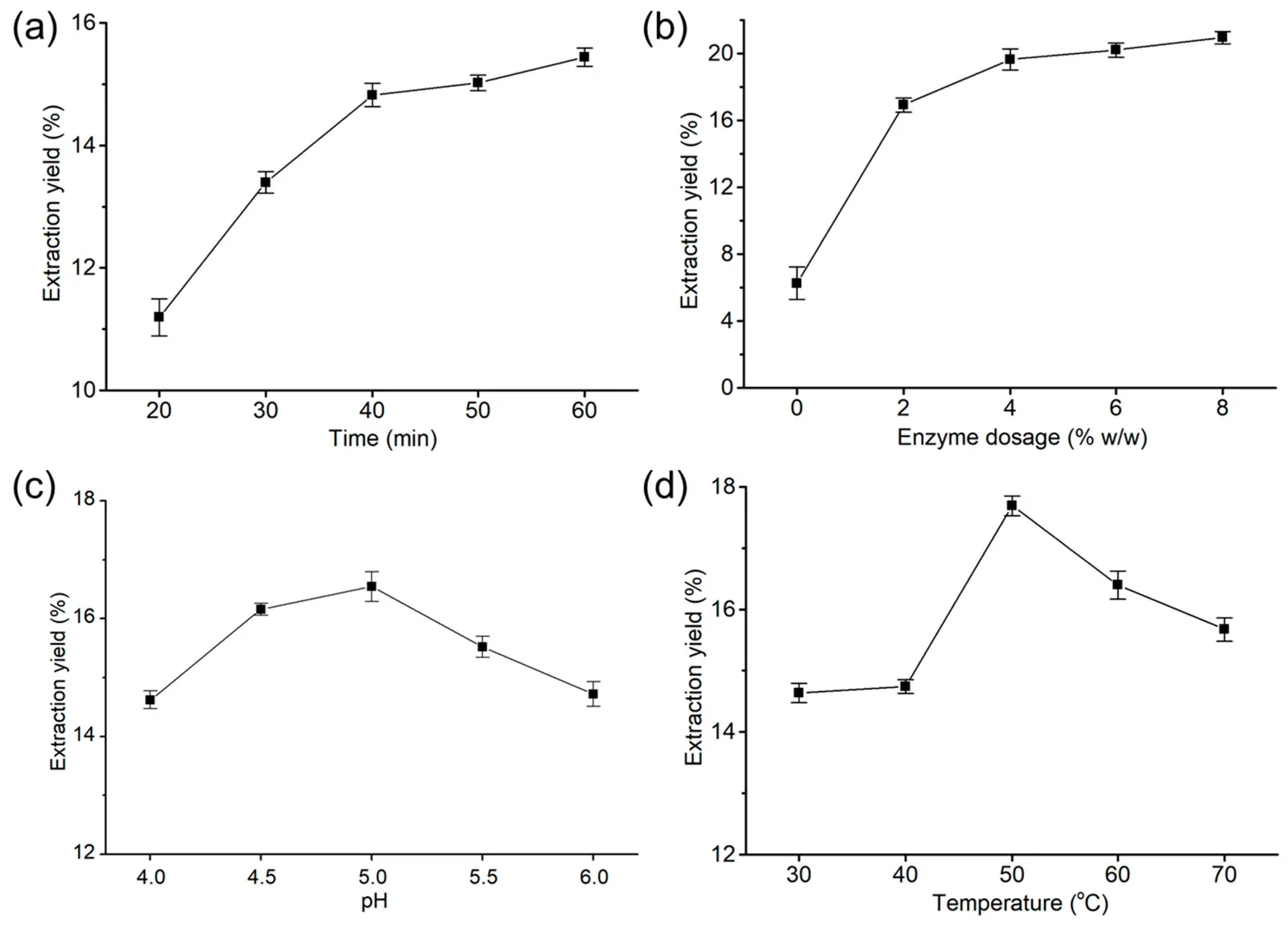
Effects of (**a**) extraction time, (**b**) enzyme dosage, (**c**) pH, and (**d**) extraction temperature on the extraction yield. Values are presented as the mean ± standard deviation of three replicates.

The effect of extraction time on extract yield is shown in Figure 1a. The yield increased markedly from 11.19 ± 0.30% at 20 min to 14.82 ± 0.19% at 40 min, indicating that prolonged extraction initially promoted the release of water-soluble materials from the root matrix. However, when the extraction time was extended beyond 40 min, only marginal increases were observed (15.02 ± 0.13% at 50 min and 15.44 ± 0.15% at 60 min), suggesting that the extraction process reached a plateau. Accordingly, an extraction time of 40 min was considered sufficient to achieve near-complete extraction.

#### 2.1.3. Effect of Enzyme Dosage on Extraction Yield

As cellulase gave the highest extraction yield in the enzyme-screening experiment, it was selected for enzyme dosage optimization. As depicted in Figure 1b, increasing the cellulase dosage from 0% (w/w) to 4% (w/w; 8.0 × 10^4^ U/g) improved the crude extract yields from 6.26 ± 0.97% to 19.65 ± 0.63%. However, a further increase to 8% (w/w; 1.6 × 10^5^ U/g) produced only a small additional increase to 20.21 ± 0.42%, which indicates that enzymatic hydrolysis may have reached equilibrium within the reaction time (extraction time). A moderate enzyme dosage was therefore preferable for balancing yield improvement and enzyme usage.

#### 2.1.4. Effect of pH on Extraction Yield

As shown in Figure 1c, the extraction yield increased from 14.62 ± 0.16% at pH 4.0 to 16.55 ± 0.24% at pH 5.0 and then declined at higher pH values. This trend is consistent with the pH-dependent nature of cellulase activity: changes in proton concentration alter the ionization states of catalytic residues and affect the overall conformation of the enzyme, thus modulating its affinity for the substrate [26]. The maximum observed at pH 5.0 may therefore reflect a more favorable environment for cellulase-mediated disruption of the cell wall matrix.

#### 2.1.5. Effect of Extraction Temperature on Extraction Yield

As illustrated in Figure 1d, raising the extraction temperature from 30 °C to 50 °C increased the extraction yield from 14.64 ± 0.15% to 17.69 ± 0.17%, whereas further heating to 70 °C reduced the yield. Moderate heating can accelerate enzymatic hydrolysis and diffusion of intracellular substances from plant cells, but higher temperature may reduce cellulase activity. Thus, selecting an appropriate extraction temperature is necessary for maximizing yield.

### 2.2. Optimization of Extraction Conditions

Based on the results of single-factor experiments, and the properties of enzyme reactions, pH, temperature and cellulase dosage were selected as the key factors for optimization of the extraction conditions. Three levels of each factor were evaluated (Table 1), and an orthogonal L_9_ (3^3^) design was employed to optimize the combination of conditions for enhancing the extraction yields.

As shown in Table 2, extraction yields obtained under various experimental conditions were analyzed by both range analysis and analysis of variance (ANOVA) to evaluate the relative influence of each factor. Range analysis indicated the following order of influence: pH > cellulase dosage > temperature. In addition, ANOVA results (Table 3) showed that both pH and cellulase dosage had a highly significant effect on extraction yield (*p* < 0.001), whereas temperature was not significant within the tested range (*p* = 0.404). These findings are consistent with the range analysis, confirming that pH and enzyme dosage are the critical parameters for optimizing the extraction process. The relatively low impact of temperature may be attributed to the narrow range of levels tested or to potential interactions with other factors. Therefore, the optimal conditions based on range analysis were identified as pH 4.5, an extraction temperature of 60 °C and a cellulase dosage of 6% (w/w; 1.2 × 10^5^ U/g).

Confirmation experiments were further performed under the optimal conditions, achieving an extraction yield of 21.59 ± 0.33%, higher than the yields in the orthogonal array. Notably, under the same optimized conditions, extraction without ultrasonication or without cellulase gave yields of 10.14 ± 0.27% and 10.92 ± 0.62%, respectively, confirming the important contributions of both the enzyme and ultrasound to the EAUE process. Similar enhancements with EAUE have also been reported for other plant materials. For example, Sánchez-Madrigal et al. reported the EAUE increased the polysaccharide extraction yield from *Dasylirion wheeleri* from 47.37% using conventional extraction at 85 °C to 77.59% with EAUE [27]. Similarly, Sun et al. reported that the extraction yield of *Polygonatum sibiricum* polysaccharide increased from 5.83% using ultrasound-assisted extraction to 43.61% using EAUE [28]. These findings highlight the great potential of EAUE for plant-waste valorization.

The phenol–sulfuric acid assay indicated that the crude extract contained 75.09 ± 0.05% total carbohydrates. High-performance gel-permeation chromatography (HPGPC) analysis revealed a heterogeneous molecular-weight distribution, comprising one component with an apparent molecular weight of 11.2 kDa and at least two components in the range of 1–2 kDa (Figure S1). Therefore, the crude extract was classified as a polysaccharide-rich extract and was further characterized by FT-IR spectroscopy, thermogravimetric analysis (TGA) and monosaccharide analysis.

### 2.3. Characterization of A. manihot Root Extract

#### 2.3.1. FT-IR Spectroscopy of Polysaccharide-Rich Extract

**Figure 2:**
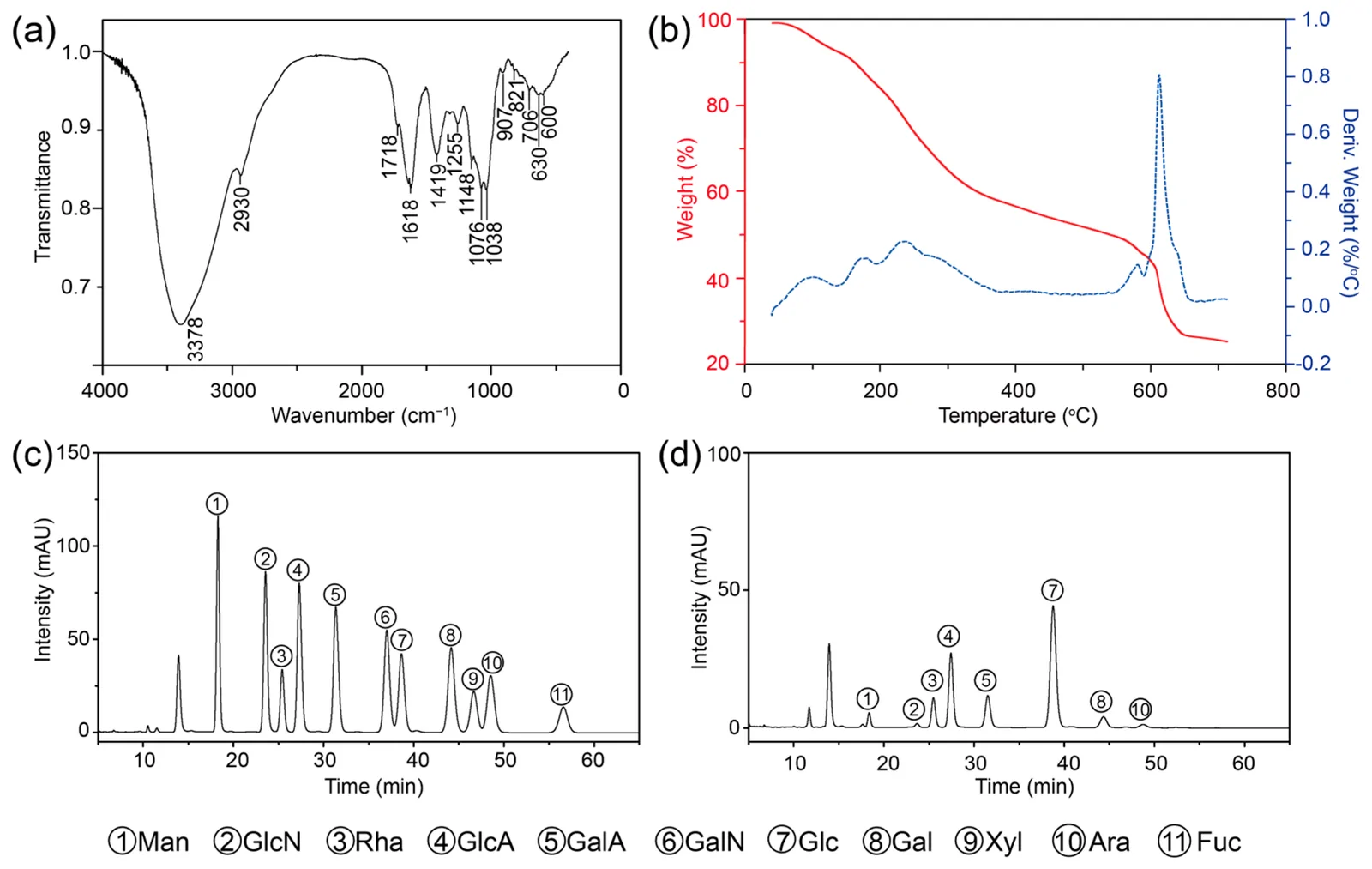
(**a**) FT-IR spectrum and (**b**) TGA (red line) and DTG (blue line) curves of the *A. manihot* root polysaccharide-rich extract. (**c**) HPLC trace of monosaccharide standards. (**d**) HPLC trace of the derivatized acid hydrolysate.

FT-IR spectroscopy is a rapid and efficient technique that can provide structural information about polysaccharide fractions [29]. As shown in Figure 2a, a strong and broad band at 3378 cm^−1^ can be attributed to the stretching vibration of abundant –OH groups in polysaccharides, while the moderate band at 2930 cm^−1^ corresponds to C–H stretching from methylene and methine groups in the carbohydrate backbone. In addition, absorption peaks at 1618 cm^−1^ and 1419 cm^−1^ can be assigned to the stretching vibrations of C=O and C–O bonds from carboxylate groups, respectively, indicating the presence of uronic acid residues in the polysaccharide components (GlcA and GalA, vide infra). Additionally, the region between 1000 and 1200 cm^−1^ is commonly considered as the “fingerprint” region of polysaccharides. The absorption peaks in this region indicate the presence of C–O–C and C–O–H stretching vibrations of pyranose rings and glycosidic linkage [30]. The absorption bands below 800 cm^−1^ are related to the carbohydrate skeletal vibrations [31]. These features support the presence of polysaccharide components in the *A. manihot* root extract, but the contributions from phenolics, salts or other co-extracted constituents to the spectrum cannot be excluded.

#### 2.3.2. TGA of *A. manihot* Root Extract

The TGA and derivative thermogravimetric (DTG) curves of the polysaccharide-rich extract demonstrate a multistep thermal degradation behavior (Figure 2b). Three distinct DTG peaks are observed at approximately 100 °C, 183 °C, and 256 °C. The mass loss occurring between 30 °C and 200 °C is about 14%, which can be attributed to the removal of physically adsorbed free water and bound water. As the temperature further increases from 200 °C to 600 °C, a pronounced mass loss of approximately 40% is observed, mainly resulting from the degradation of the carbohydrate through dehydroxylation and deoxygenation processes [32]. The substantial mass loss above 600 °C is associated with the decomposition or oxidation of carbonaceous residues formed during earlier pyrolysis stages. Overall, these results indicate that the *A. manihot* root extract possesses moderate thermal stability and undergoes degradation via multiple thermal events.

#### 2.3.3. Composition Analysis of Monosaccharides

The monosaccharide composition of the hydrolyzed *A. manihot* root extract was estimated by comparing the HPLC chromatograms of the hydrolysate with those of monosaccharide standards (Figure 2c,d). The detected derivatives corresponded mainly to glucose (Glc, 49.6 mol%), glucuronic acid (GlcA, 16.1 mol%), rhamnose (Rha, 15.9 mol%), and galacturonic acid (GalA, 8.67 mol%), with smaller proportions of galactose (Gal, 4.9 mol%), mannose (Man, 2.3 mol%), and arabinose (Ara, 1.7 mol%). This profile is broadly consistent with previous reports on *A. manihot* polysaccharide fractions [5,11,33]. For example, Zheng et al. reported that AMPS-a, a polysaccharide isolated from the flowers of *A. manihot*, was primarily composed of glucose, mannose, galactose and fucose in a molar ratio of 1.00:0.91:2.14:1.09. [34] Moreover, *A. manihot* polysaccharide fractions have been reported to exhibit radical scavenging, anti-inflammatory, and antimicrobial activities [33,34,35], which may be relevant to hair care efficacy. Therefore, the preliminary hair care-related properties of the crude extract were subsequently evaluated.

### 2.4. Evaluation of Hair Care-Related Properties

#### 2.4.1. Analysis of Antioxidant Activity

Ultraviolet (UV) exposure can generate reactive oxygen species or free radicals that contribute to chemical damage in hair fibers [36], and antioxidants can mitigate the damage by preventing protein degradation and lipid peroxidation in hair [37]. Therefore, the antioxidant capacity of the extract is considered an important factor contributing to its potential hair care effect.

**Figure 3:**
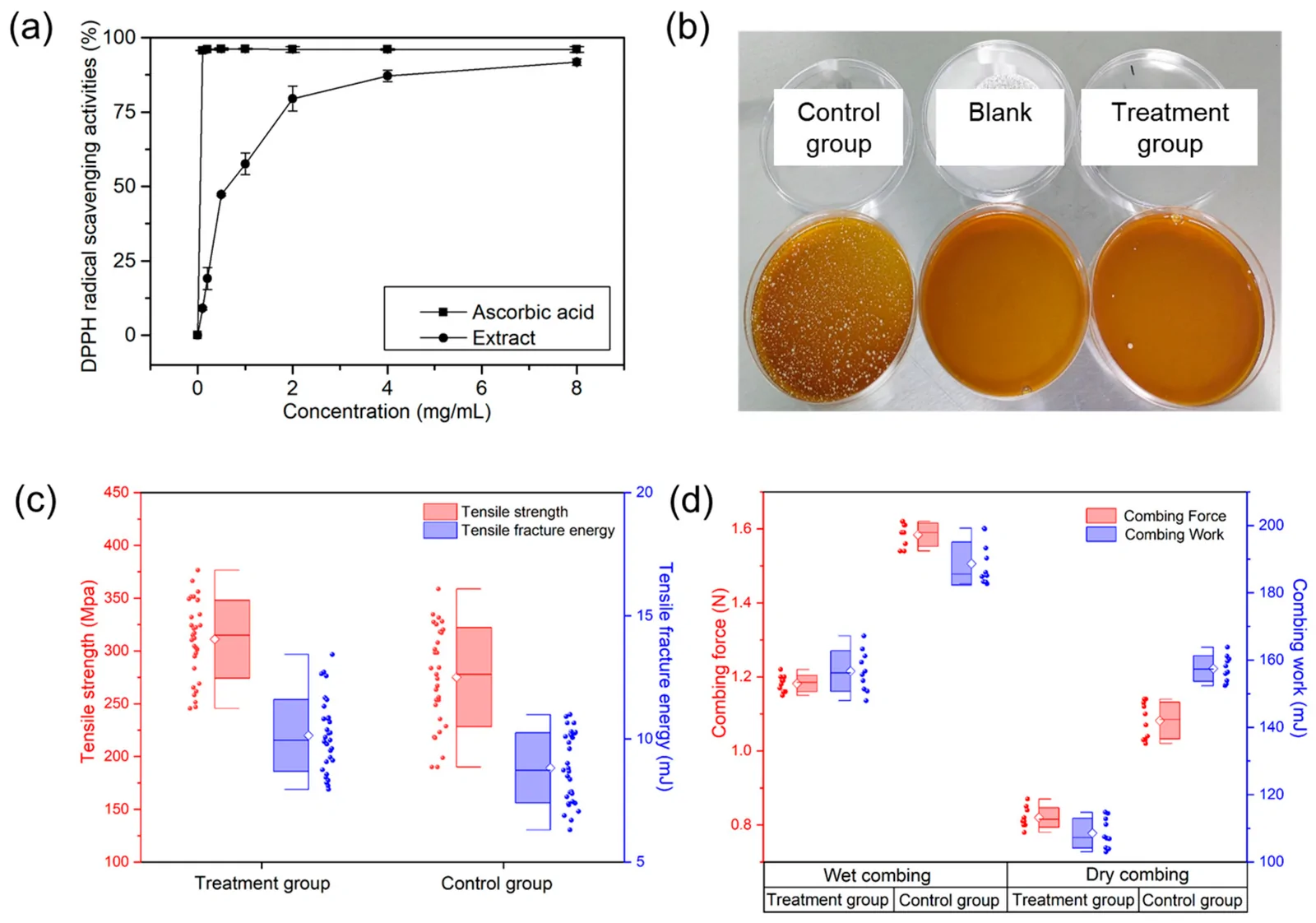
(**a**) DPPH free radical scavenging activity of the polysaccharide-rich extract compared with the ascorbic acid as a reference. (**b**) Growth of *Malassezia furfur* in the control, blank and extract treatment groups. The uninoculated blank verified the absence of contamination during the experimental procedure. (**c**) Box and whisker plot of tensile strength (red) and tensile fracture energy (blue) in the treatment and control groups. (**d**) Box and whisker plot of wet and dry combing forces (red) and combing work (blue) in the treatment and control groups. Boxes show the interquartile range, the center line indicates the median, diamonds indicate the mean, and whiskers show the minimum and maximum.

To evaluate the antioxidant activity of the *A. manihot* root extract, the 1,1-diphenyl-2-picryhydrazyl (DPPH) radical scavenging assay was conducted (Figure 3a). The results showed that the DPPH radical scavenging efficiency of the polysaccharide-rich extract increased significantly from 9.00% to 79.52% over the concentration range of 0.1–2.0 mg/mL, indicating a clear dose-dependent antioxidant effect. Further elevation of the concentration from 2.0 to 8.0 mg/mL led to a comparatively smaller increase, with the radical scavenging efficiency reaching 91.75%. For comparison, ascorbic acid exhibited exceptionally strong radical scavenging ability, achieving 95.68% efficiency even at a low concentration of 0.1 mg/mL. Although the antioxidant efficiency of *A. manihot* root extract was lower than that of ascorbic acid at low concentrations, it nevertheless exhibited substantial radical scavenging efficiency at concentrations above 2.0 mg/mL. These findings demonstrated the antioxidant capacity of the extract under the specified assay conditions, which may be relevant to mitigating oxidative processes associated with UV-induced hair damage. However, the DPPH assay does not directly establish protection of hair fibers against UV exposure. Further experiments using UV-exposed hair models are required to confirm the protective efficacy of the extract and elucidate the underlying mechanisms.

#### 2.4.2. Evaluation of Anti-Dandruff Efficacy

*M. furfur* is a lipophilic yeast commonly found on the human skin surface. Its abnormal proliferation has been closely associated with skin diseases such as seborrheic dermatitis and dandruff [11]. Therefore, antifungal activity against *M. furfur* is a key indicator for assessing anti-dandruff efficacy. As shown in Figure 3b, the *A. manihot* root extract at its saturated concentration (30 mg/mL) inhibited *M. furfur* growth by 84%, indicating its antifungal potential. Although polysaccharides have been reported to possess antimicrobial activity, the extract also contained 1.65 ± 0.03% total flavonoids (please refer to Supplementary Material for details), some of which, such as quercetin and isoquercetin may also contribute to the antifungal activity against *M. furfur* [6,38]. Consequently, the principal antifungal constituents cannot yet be conclusively identified, and further fractionation and component-specific studies are required to clarify the antifungal mechanism.

#### 2.4.3. Assessment of Anti-Breakage Efficacy

The mechanical properties of hair fibers play an important role in determining hair appearance, manageability and overall health. To evaluate the protective effect of the polysaccharide-rich extract, tensile strength and tensile fracture energy were measured as indicators of hair strength, elasticity and resistance to mechanical damage. Compared to the control group, treatment with the extract significantly enhanced the average tensile strength from 275.2 MPa to 311.09 MPa, and increased the average tensile fracture energy from 8.83 mJ to 10.14 mJ (Figure 3c). These improvements demonstrate the anti-breakage efficacy of the polysaccharide-rich extract.

#### 2.4.4. Evaluation of Hair Combability

Hair combability is a critical parameter in hair care, as poor combability can result in increased friction, tangling, and even mechanical breakage during combing. To quantitatively assess hair combability, both combing force and combing work under wet and dry conditions were measured, reflecting the mechanical resistance encountered when a comb passed through hair fibers. Lower values of combing force or combing work indicate reduced resistance and thus improved hair combability.

As illustrated in Figure 3d, treatment with *A. manihot* root extract solution at 30 mg/mL significantly enhanced hair combability. Specifically, the wet combing force decreased from 1.58 N to 1.18 N, while the dry combing force declined from 1.08 N to 0.82 N. Correspondingly, the wet and dry combing work of the treatment group decreased by 16.93% (from 188.67 mJ to 156.73 mJ) and 30.86% (from 157.47 mJ to 108.57 mJ), respectively, compared to the control group. These results demonstrate the effectiveness of *A. manihot* root extract in enhancing hair manageability and facilitating detangling.

#### 2.4.5. Possible Surface-Conditioning Interpretation

**Figure 4:**
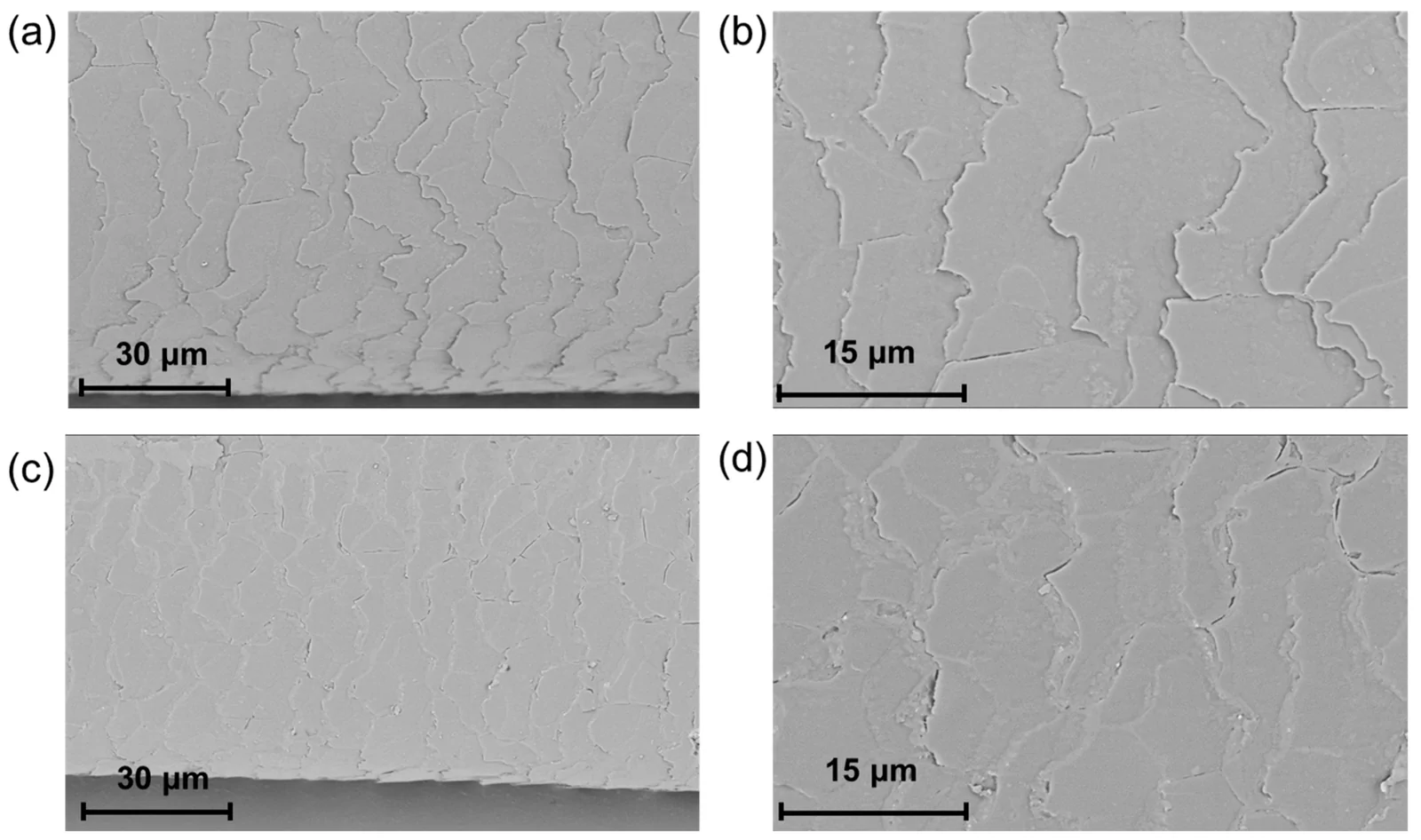
SEM images showing the surface morphology of hair fibers in the control group (**a**,**b**) and the polysaccharide-rich extract-treated group (**c**,**d**) at different magnifications.

SEM was employed to examine the surface morphology of hair fibers before and after treatment with the *A. manihot* root extract. As illustrated in Figure 4a,b, damaged hair often exhibits lifted cuticles and surface roughness, which increase inter-fiber friction. However, the extract-treated hair fibers appeared smoother, with fewer prominently lifted cuticle edges (Figure 4c,d) [39]. Similar phenomena were also observed after treatment with other plant extracts. For example, Benaiges et al. reported that treating bleached hair with *Cystoseira compressa* seaweed extract, *Lepidium meyenii* extract or *carob tree* extract could reduce cuticle lifting, smooth the fiber surface, and exert a protective effect on the hair [40]. Additionally, the hydrophilic polysaccharides can enhance hair moisture retention and improve the softness and elasticity of the hair fiber [41]. These effects collectively reduce mechanical resistance and improve the overall ease of combing [42].

Human hair consists predominantly of keratin, and disulfide crosslinks contribute to its mechanical strength and stability [43]. Based on SEM observations, two non-exclusive explanations for the observed changes are proposed:

1. Moisture retention: Hydrophilic constituents in the extract may retain water at the hair surface or in damaged cuticle regions, which could reduce brittleness and increase flexibility [40].
2. Surface deposition: Film-forming constituents may adsorb to hair fibers, smooth lifted cuticle edges, reduce inter-fiber friction, and redistribute stress during tensile deformation [40].

## 3. Materials and Methods

### 3.1. Materials and Reagents

Air-dried *A. manihot* roots were obtained from Suzhong Pharmaceutical Group Co., Ltd. (Taizhou, China). The root material was ground into fine powder and subsequently passed through an 80-mesh sieve (pore size = 0.6 mm) prior to use. DPPH (≥98.5%), pectinase (500 U/mg, P758970), cellulase (400 U/mg, C758963) and papain (200 U/mg, P6321) were purchased from Macklin Biochemical Technology Co., Ltd. (Shanghai, China). HPLC-grade solvents and commercial standards for HPLC analysis were purchased from Adamas Reagent Co. Ltd. (Shanghai, China) and used without further purification. Ultrapure water was obtained from a Master-S UP laboratory water purification system (HHitech, Shanghai, China).

### 3.2. Enzyme-Assisted Ultrasonic Extraction for Polysaccharides

#### 3.2.1. General Method of EAUE

*A. manihot* root powder (5.0 g) was weighed and mixed with ultrapure water at a solid-to-liquid ratio of 1:20 (g/mL), followed by overnight soaking at room temperature (~25 °C) to hydrate the dried root matrix before enzymatic and ultrasonic treatment. After soaking, the pH of the mixture was adjusted to the designed value using 4 M HCl and 2.8 M NaOH and the enzyme was added. EAUE was conducted in continuous mode using an ultrasonic bath (KQ-250DE, Kunshan ultrasonic Instruments Co., Ltd., Suzhou, China) operating at a power output of 250 W and a frequency of 40 kHz, under designed time and temperature conditions. After rapid suppressing of enzyme activity in ice water for 15 min, the sample was centrifuged at 6000 rpm for 5 min. Then the supernatant was collected and deproteinized using the Sevag method. The resulting solution was then lyophilized to obtain the *A. monihot* root extract. The extraction yield (*Y*) was calculated using the following equation:

(1) $$Y\left(\%\right)=\frac{m_{E}}{m_{A}}\times 100\%$$

where *m_E_* represents the mass of polysaccharide-rich extract, and *m_A_* is the initial mass of *A. manihot* root powder.

#### 3.2.2. Condition Optimization

The enzyme type was initially screened by comparing pectinase, cellulase, and papain at an enzyme dosage of 1% (w/w, relative to dry root powder), a solid-to-liquid ratio of 1:20 (g/mL), pH 5.0, an extraction temperature of 40 °C, and an ultrasonication time of 40 min. Cellulase was subsequently used in the single-factor experiments. The baseline conditions were a solid-to-liquid ratio of 1:20 (g/mL), a cellulase dosage of 1% (w/w), pH 5.0, an extraction temperature of 40 °C, and an ultrasonication time of 40 min. When one parameter was evaluated, it was varied over the selected range while all other conditions were maintained at their baseline values. The investigated ranges were 0–8% (w/w) cellulase (0–1.6 × 10^5^ U/g relative to dry root powder), extraction temperature 30–70 °C, pH 4.0–6.0, and extraction time 20–60 min. Each extraction was independently performed three times. Based on the results of the single-factor experiments, an orthogonal experimental design (L_9_ (3^3^)) was conducted under selected conditions, and range analysis was performed to determine the optimal extraction parameters.

### 3.3. Characterization of A. manihot Root Extracts

#### 3.3.1. Determination of Total Carbohydrate Content

The total carbohydrate content of the extract was determined by the phenol–sulfuric acid method. Briefly, a series of glucose standard solutions (1.0 mL) were treated with 1.0 mL of a freshly prepared 5% (w/v) phenol solution and 5.0 mL of concentrated sulfuric acid. The mixture was heated for 20 min and the absorbance was measured at 490 nm to generate a calibration curve. The extract solution (0.1 mg/mL) was analyzed under the same conditions, and the polysaccharide content was calculated based on the calibration curve.

#### 3.3.2. FT-IR Measurement

The polysaccharide-rich extract sample was thoroughly mixed with spectroscopic-grade potassium bromide and ground into a fine powder. The mixture was then compressed into a translucent pellet for infrared analysis. The FT-IR spectrum was recorded using a Thermo Scientific Nicolet IS20 FT-IR spectrometer (Thermo Fisher Scientific Inc., Waltham, MA, USA) over the wavenumber range of 500–4000 cm^−1^.

#### 3.3.3. TGA Measurement

The thermal stability of the obtained polysaccharide extract was evaluated by TGA using a Discovery TGA 550 instrument (TA Instruments, New Castle, Germany). Measurements were carried out over a temperature range of 40–720 °C at a constant nitrogen flow of 50 mL/min and a heating rate of 10 °C/min. Weight loss was calculated relative to the initial sample mass, and the resulting thermogravimetric data were analyzed using TRIOS software (V6.1, TA Instruments, New Castle, Germany).

#### 3.3.4. Analysis of Monosaccharide Composition

Monosaccharide composition analysis was performed using a previously reported 1-phenyl-3-methyl-5-pyrazolone (PMP) pre-column derivatization method. A total of 5 mg of the polysaccharide sample was hydrolyzed in 1 mL of 2 mol/L trifluoroacetic acid at 120 °C for 2 h, and the hydrolysate was dried, washed with methanol and diluted in 1 mL of ultrapure water for analysis. Then a mixed monosaccharide standard solution containing arabinose (Ara), fucose (Fuc), galactose (Gal), glucose (Glu), mannose (Man), rhamnose (Rha), xylose (Xyl), galacturonic acid (GalA), glucuronic acid (GlcA), galactosamine hydrochloride (GalN), and glucosamine hydrochloride (GlcN) was prepared by serial dilution to final concentrations ranging from 10 to 1000 μg/mL. To enhance UV absorbance and improve chromatographic separation, 1-Phenyl-3-methyl-5-pyrazolone (PMP) derivatization was applied. Specifically, 0.2 mL of hydrolyzed sample or standard solution was mixed with 0.2 mL of 0.5 mol/L NaOH and 0.5 mL of 0.5 mol/L PMP solution and allowed to react at 70 °C for 1 h. The reaction mixture was then neutralized with HCl and washed with chloroform to remove excess PMP. The aqueous phase was diluted to 1.0 mL with water for HPLC analysis. Chromatographic separation was performed using an UltiMate 3000 HPLC system (Thermo Fisher Scientific Inc., Waltham, MA, USA) equipped with an Agilent ZORBAX Eclipse XDB-C18 column (4.6 × 250 mm, 5 μm; Agilent Technologies, Inc., Santa Clara, CA, USA) with a mobile phase of phosphate buffer (pH 6.8) and acetonitrile at a ratio of 17/83 (v/v) and a flow rate of 0.8 mL/min. The column temperature was maintained at 30 °C and detection was carried out at 250 nm. Monosaccharides were identified and quantified by comparing retention times and peak areas with those of the corresponding standards.

### 3.4. Hair Care Efficacy Evaluation

#### 3.4.1. Antioxidant Activity Assay

A total of 1.0. mL of the diluted extract solution (0.1–8 mg/mL in H_2_O) was treated with 1.0 mL of DPPH solution (0.1 mM). The mixture was incubated at 25 °C for 30 min, followed by UV-vis spectroscopic measurement at 517 nm. The control and reference groups were prepared using 1 mL of deionized H_2_O and a series of ascorbic acid solutions under the same treatment conditions, respectively. Each measurement was performed three times independently. *DPPH radical scavenging activity* (%) was calculated according to the following equation:

(2) $$DPPH\;radical\;scavenging\;activity\;\left(\%\right)=\left(1-\frac{A_{sample}}{A_{control}}\right)\times 100\%$$

where *A_sample_* and *A_control_* are the absorbances of the sample group and control groups at 517 nm.

#### 3.4.2. Anti-Dandruff Efficacy Evaluation

To evaluate the anti-dandruff potential of the *A. manihot* polysaccharide extract, an antifungal assay was conducted following a method based on Chinese Industry Standard QB/T 2738-2012 [44], with the assistance of CAS testing technical services (Guangzhou) Co., Ltd. In brief, the *M. furfur* suspension was diluted to approximately 1 × 10^5^ CFU/mL with sterile phosphate-buffered saline, as determined using the standard viable plate-count method. The suspension was then combined either with extraction solution (treatment group, 30 mg/mL) or with phosphate buffer (control group). Each mixture was then incorporated into molten agar-based nutrient medium. Upon solidification, the plates were inverted and incubated for 5 days at 35 °C. Following incubation, colony-forming units were enumerated, and the inhibition rate (*IR*) of *M. furfur* growth was calculated using the following equation:

(3) $$IR\left(\%\right)=\frac{A-B}{A}\times 100\%$$

where *A* represents the average colony-forming units of the control group and *B* denotes the average colony-forming units of the treatment group.

#### 3.4.3. Hair Combability Test

Hair care efficacy evaluation was performed with the assistance of CAS testing technical services (Guangzhou) Co., Ltd. The hair conditioning efficacy of the *A. manihot* root extract was evaluated via combability tests based on the guidelines of Chinese Group Standard T/GDCA017-2022 [45]. Specifically, ten standardized hair tresses (~6 g per tress) were randomly assigned to a treatment group (treated with polysaccharide solution) and a control group (treated with deionized water). Based on our previous study [11], a concentration of 30 mg/mL (the approximate saturated solubility of the polysaccharide) was selected for further research. All samples were thoroughly wetted and treated with a 10% (w/v) sodium dodecyl sulfate surfactant solution for 1 min. After rinsing for 30 s, the wet combing forces and combing work baseline of each tress were measured and recorded using a fibra.one multi-function hair tress testing instrument (Dia-Stron Limited, Andover, UK). The tresses were then air-dried in a constant temperature and humidity chamber for 4 h. For the tests, the tresses in the treatment group were subsequently treated with the *A. manihot* root extract solution for 1 min and rinsed for 30 s, while the control group received the same treatment using only water. Post-treatment wet and dry combing forces and combing work were then measured following the same procedures as those used for baseline measurements.

#### 3.4.4. Anti-Breakage Efficacy Evaluation

To simulate damaged hair tresses, cleaned hair samples were treated with a mixed solution of 1% hydrogen peroxide and 2% ammonia at 40 °C for 2 h. Following treatment, the hair was rinsed with ultrapure water, neutralized in a 10 g/L citric acid solution for 5 min, and subsequently air-dried under ambient conditions (temperature 26 ± 2 °C, relative humidity 60 ± 10%) for 24 h. The diameter of each hair tress was measured using a micrometer for tensile strength and tensile fracture energy measurements. The anti-breakage efficacy of *A. manihot* root extract solution (30 mg/mL) was assessed according to the Chinese Group Standard T/GDCDC012-2020 [46]. For the treatment group, hair tresses were treated with polysaccharide-rich extract solution (0.2 g/g) for 30 min, while the control group was treated with water under the same conditions. After rinsing, all tresses were air-dried under the same ambient conditions for an additional 24 h. The tensile force and tensile fracture energy of each tress were measured using a hair tress testing instrument. The tensile strength (*TS*) was calculated as follows:

(4) $$TS(\mathrm{MPa})=\frac{TF}{A}$$

where *TF* is the tensile force (N) and *A* represents the cross-sectional area of the hair tress (mm^2^).

#### 3.4.5. Scanning Electron Microscopy (SEM) Measurement

The surface morphology and cuticle structure of hair fibers were imaged using a YB-200E SEM instrument (Phenom Scientific Instrument Co., Ltd., Shanghai, China) at an accelerating voltage of 5 kV. Prior to imaging, the hair samples were sputter-coated with a thin gold layer using a KYKY SBC-12 ion sputter coater (KYKY technology Co., Ltd., Beijing, China) to enhance electrical conductivity and ensure image quality.

### 3.5. Statistical Analysis

Unless otherwise stated, extraction experiments were independently performed three times and the results are presented as the mean ± standard deviation. ANOVA was performed using SPSS 26.0 software, with *p* < 0.05 considered statistically significant. Sample sizes for hair tress and hair fiber assays were analyzed and visualized using Origin 2021 software.

## 4. Conclusions

This study highlights the effectiveness of EAUE in enhancing the extraction yield of the polysaccharide-rich extract from *A. manihot* root by facilitating the degradation of root cell walls and promoting the release of intracellular metabolites. Compared with extraction without ultrasonication (10.14%) or enzyme assistance (10.92%), the optimized process achieved a significantly higher extraction yield of 21.59%, underscoring the efficiency of this approach. Furthermore, the potential application of this polysaccharide-rich extract in hair care was preliminarily demonstrated. The extract solution (30 mg/mL) exhibited significant antifungal activity (84% inhibition rate), improved hair combability (reducing wet and dry combing force and work by 16.93–30.86%), and enhanced resistance to mechanical breakage (increasing tensile strength and tensile fracture energy by 13.04% and 14.84%, respectively), indicating its promising efficacy in anti-dandruff treatment, hair manageability and hair strength maintenance. In summary, this work contributes to sustainable agriculture and green chemistry by valorizing *A. manihot* root—a commonly discarded agricultural byproduct—while broadening its application in hair care products. Future research should focus on elucidating the structure–activity relationship between polysaccharide molecules and hair care effects and exploring its practical applications in hair care cosmetic products.

## Figures and Tables

**Table 1:** Factors and levels selected for extraction condition optimization.

Level	Factors		
	A: pH	B: Temperature (°C)	C: Enzyme Dosage (% w/w) ^1^
1	4.5	40	2
2	5.0	50	4
3	5.5	60	6

^1^ Enzyme (cellulase) dosages of 2% (w/w), 4% (w/w), and 6% (w/w; relative to dry root powder) corresponded to activities of 4.0 × 10^4^ U/g, 8.0 × 10^4^ U/g, and 1.2 × 10^5^ U/g of dry root powder, respectively.

**Table 2:** Orthogonal experiment for condition optimization. ^1^

Entry	pH	Temperature (°C)	Enzyme Dosage (% w/w)	Extraction Yield ^2^ (%)
1	4.5	40	2	18.82 ± 1.27
2	4.5	50	6	20.83 ± 0.29
3	4.5	60	4	19.34 ± 0.25
4	5.0	40	6	19.55 ± 0.19
5	5.0	50	4	17.54 ± 0.46
6	5.0	60	2	18.36 ± 1.05
7	5.5	40	4	17.41 ± 0.98
8	5.5	50	2	17.03 ± 0.51
9	5.5	60	6	19.10 ± 0.89
K_1_	19.66	18.59	18.07	
K_2_	18.48	18.47	18.09	
K_3_	17.85	18.93	19.82	
R	1.81	0.46	1.75	

^1^ K represents the mean value of the experimental results corresponding to a particular level of a specific factor; ^2^ the extraction yields are expressed as the mean ± standard deviation of three replicates.

**Table 3:** Variance analysis of orthogonal test.

Factors	Sum of Squares	Degrees of Freedom	Mean Square	F-Value	*p*-Value
pH	15.29	2	7.65	14.03	<0.001 ***
Temperature	1.05	2	0.52	0.96	0.40
Cellulase dosage	18.24	2	9.12	16.73	<0.001 ***
Error	10.90	20	0.55		
Total	45.48	26			

Note: *** *p* < 0.001.

## Data Availability

The datasets supporting this article are included in the manuscript.

## Supplementary Materials

The following supporting information can be downloaded at: https://www.mdpi.com/article/10.3390/molecules31162817/s1, Figure S1: HPGPC chromatogram of the *A. manihot* root extract. Note: The peak at approximately 42.2 min corresponds to the mobile-phase salt peak; Table S1: Molecular weight distribution of the *A. manihot* root extract determined by HPGPC; Figure S2: Rutin calibration curve for total flavonoid quantification.
