# Supplementary material for: Enzyme-Assisted Ultrasonic Extraction of a Polysaccharide-Rich Extract from Abelmoschus manihot (L.) Root and Preliminary Evaluation of Hair Care-Related Properties
Source: Molecules. 2026 Aug 13;31(16):2817. doi: 10.3390/molecules31162817 (PMC13515090; doi:10.3390/molecules31162817)
Supplement: Supplementary file 1 [file molecules-31-02817-s001.zip › molecules-4455435-supplementary.pdf]

Supplementary Information

Enzyme-Assisted Ultrasonic Extraction of a Polysaccharide-Rich Extract from *Abelmoschus manihot* (L.) Root and Preliminary Evaluation of Hair Care-Related Properties

Junjie Wang [1,2,†], Xueyan Liu [2,3,†], Dian Zhuang [2,3], Zixuan Ren [2], Weihong Chen [1], Mingqiong Guo [1], Zenglai Xu [2,*] and Qiong Wang [2,*]

[1] Department of Chemistry, School of Science, Xihua University, Chengdu 610039, China; jjwang@xhu.edu.cn (J.W.); chenweihong@stu.xhu.edu.cn (W.C.); guomingqiong@stu.xhu.edu.cn (M.G.)
[2] Jiangsu Key Laboratory for Conservation and Utilization of Plant Resources, Institute of Botany, Jiangsu Province and Chinese Academy of Sciences (Nanjing Botanical Garden Memorial Sun Yat-Sen), Nanjing 210014, China; xueyan@njucm.edu.cn (X.L.); zdmm126com@126.com (D.Z.); rzx961838706@gmail.com (Z.R.)
[3] Nanjing University of Chinese Medicine, Nanjing 210023, China
* Correspondence: xuzenglai@jib.ac.cn (Z.X.); wangqiong@cnbg.net (Q.W.)
† These authors contributed equally to this work.

1. High-performance gel permeation chromatography (HPGPC) of the polysaccharide-rich extract

Method: The molecular-weight distribution of the extract was determined by high-performance gel permeation chromatography (HPGPC) using a Waters 1515 HPLC system equipped with a Waters 2414 refractive index detector and a Waters 2707 autosampler. Two aqueous SEC columns (Ohpak SB-804 HQ and Ohpak SB-806M HQ, 8 × 300 mm) were connected in series. A series of dextran standards with known molecular weights was used to construct calibration curves by plotting the logarithm of molecular weight against retention time. The extract (5 mg) was dissolved in 1 mL of 0.05 M NaCl solution, centrifuged at 8000 rpm for 10 min, and filtered through a 0.22 μm membrane before analysis. The chromatographic separation was performed using 0.05 M NaCl as the mobile phase at a flow rate of 0.50 mL/min, a column temperature of 40 °C, and an injection volume of 30 μL.

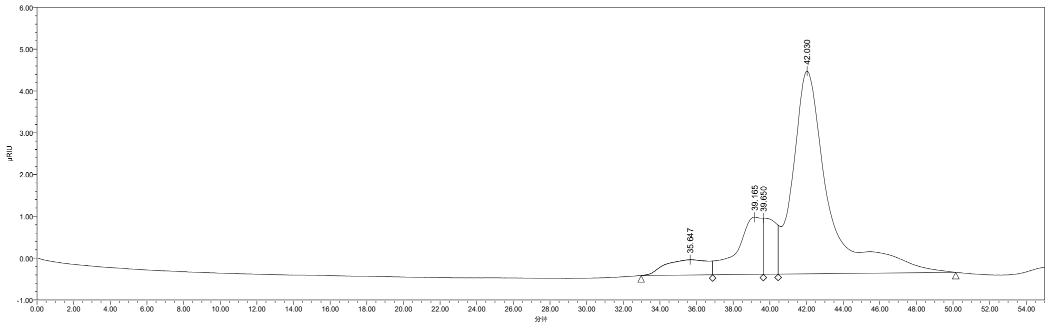

Figure S1. HPGPC chromatogram of the *A. manihot* root extract. Note: The peak at approximately 42.2 min corresponds to the mobile-phase salt peak.

Table S1. Molecular weight distribution of the *A. manihot* root extract determined by HPGPC.

**Retention time (min)**	**Mp（Da）**	**Mw（Da）**	**Mn（Da）**
35.647	9583	11264	7990
39.165	1698	1826	1421
39.650	1338	1421	1120

2. Determination of the total flavonoid content of the *A. manihot* root extract

The total flavonoid content was determined using the sodium nitrite–aluminum nitrate colorimetric method with rutin as the reference standard. The crude polysaccharide extract (0.10 g) was dissolved and diluted to 50 mL, and a 5.0 mL aliquot was transferred to a 10 mL volumetric flask. Subsequently, 0.3 mL of 5% $NaNO_2$ solution was added, and the mixture was allowed to stand for 6 min. After the addition of 0.3 mL of 10% $Al(NO_3)_3$ solution, the mixture was incubated for another 6 min, followed by the addition of 4.0 mL of 4% NaOH solution. The solution was diluted to volume with 30% ethanol and allowed to stand for 15 min before its absorbance was measured at 506 nm. For calibration, rutin (10.0 mg) was dissolved in 70% ethanol and diluted to 50 mL to prepare a 200 mg/L stock solution. Aliquots of 0, 1.0, 2.0, 3.0, 4.0, and 5.0 mL of the rutin stock solution were subjected to the same color-development procedure. The total flavonoid content was calculated from the rutin calibration curve and expressed as rutin equivalents.

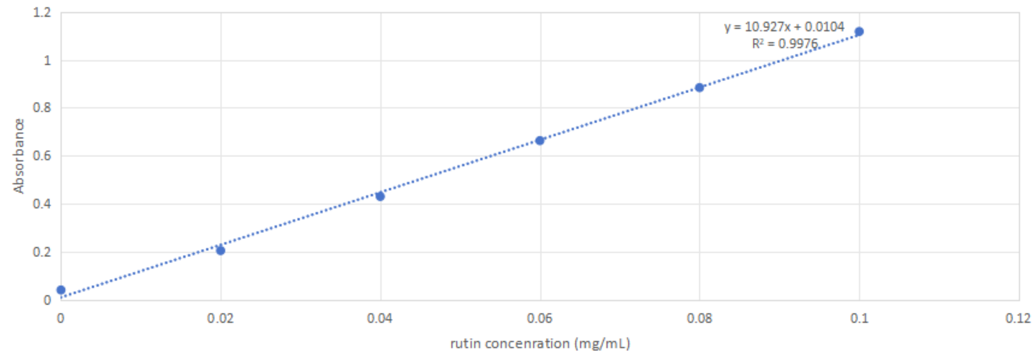

Figure S2. Rutin calibration curve for total flavonoid quantification
